# Investigation of Electrical Discharge Machining Micro Holes in CoCrFeNiZr_0.5_ Eutectic High Entropy Alloys

**DOI:** 10.3390/mi17050589

**Published:** 2026-05-11

**Authors:** Qingming Fan, Longfei Liu, Guokang Su, Chuanyun Zhang, Man Zhu, Kai Cheng

**Affiliations:** 1 School of Mechatronic Engineering, Xi’an Technological University, Xi’an 710021, China; fanqingming@xatu.edu.cn (Q.F.); llf20021102@163.com (L.L.); suguokang@xatu.edu.cn (G.S.); 2 Shaanxi Engineering Research Center of Digital Precision Electrochemical Machining, Xi’an 710021, China; 3 School of Materials Science and Chemical Engineering, Xi’an Technological University, Xi’an 710021, China; zhuman0428@126.com; 4 College of Engineering, Design and Physical Sciences, Brunel University London, London UB8 3PH, UK

**Keywords:** eutectic high-entropy alloys (EHEAs), electrical discharge machining (EDM), micro hole machining, Micro-EDM drilling, material design and manufacturing

## Abstract

As one of the most promising new materials in the field of materials science, high-entropy alloys (HEAs) have attracted widespread attention due to the unique structure, exceptional properties and engineering performance, and complex composition. The CoCrFeNiZr_0.5_ eutectic high-entropy alloys (EHEAs) exhibits excellent high-temperature thermal stability, ductility, creep resistance, and corrosion resistance, demonstrating great potential for applications in marine equipment. This paper explores the engineering feasibility of electrical discharge machining (EDM) of CoCrFeNiZr_0.5_ EHEAs and investigates the EDM of micro-holes using a hollow copper electrode on a CNC EDM drilling machine under various machining parameters, including different gap voltage, pulse-on time, pulse-off time, and pulse amplifier settings. The effects of these parameters on the inlet diameter, outlet diameter, and recast layer of the micro holes are analyzed. The optimal micro-hole machining parameters are determined by comprehensively considering machining efficiency and electrode wear: gap voltage of 33 V, pulse-on time of 3 μs, pulse-off time of 1 μs, and pulse amplifier output of 3 A. Adopting the parameters to process a button ingot sample with a depth of 5 mm, it was found that the machining speed is 7.79 mm/min and the electrode wear is 1 cm. This research renders the foundation for further development and engineering application of CoCrFeNiZr_0.5_ EHEAs in the context of high-value material design and manufacturing.

## 1. Introduction

The concept of high-entropy alloys (HEAs) was first proposed by Yeh et al. [1]. HEAs contain at least five principal elements, with each element having a concentration in the range of 5–35 at.%, which provides a new avenue for the design and manufacturing of high-value alloys. Due to their unique structure, HEAs exhibit excellent comprehensive properties, including high strength, high hardness, high-temperature oxidation resistance, superior thermal stability, and good corrosion resistance [2,3,4,5,6]. Single-phase Face-Centered Cubic (FCC) HEAs possess excellent ductility but relatively low strength, while single-phase Body-Centered Cubic (BCC) HEAs have high strength but poor ductility. To overcome the balance between high strength and high ductility, the design idea of eutectic high-entropy alloys (EHEAs) was proposed [7]. The EHEAs have the advantages of both HEAs and eutectic alloys, so they possess excellent castability, high strength, and good ductility [7,8,9]. The CoCrFeNiZr_0.5_ EHEAs are fully lamellar eutectic structure alloys with FCC + Laves phases [10]. Zirconium (Zr) is a key element in the formation of the Laves phase, which can significantly enhance the mechanical properties of the EHEAs at both room and high temperatures [11]. The CoCrFeNiZr_0.5_ EHEAs have high yield strength and better corrosion resistance than SS304 [12], which makes them have potential application prospects in industrial fields such as marine propellers and hydraulic pump rudder blades [7,9].

As one of the most promising new materials in the field of materials science, the HEAs have attracted widespread attention from researchers in disciplines such as mechanical engineering and materials science [13]. Among the currently developed HEA systems, the CoCrFeNi series has been the most extensively studied [14]. Recent research efforts have increasingly focused on designing multicomponent alloys with different compositions by adjusting the types and amounts of alloying elements or on applying heat treatments to obtain high-performance HEA materials that meet various engineering requirements. The objective is to develop novel high-performance HEAs and maximize their application potential across different fields—for example, by adding metallic elements such as Ta, Nb, Zr, Ti, Al, and Mo to the CoCrFeNi HEAs to enhance their strength [15,16]. The study by Qi et al. [12] also demonstrated that Zr can be incorporated into HEAs as a corrosion-resistant metallic element to improve corrosion resistance. However, in CoCrFeNiZr0.5 HEAs, the Zr-induced formation of an FCC + Laves dual-phase structure, along with lattice distortion and second-phase strengthening, significantly enhances strength, hardness, corrosion resistance, and wear resistance. This is accompanied by a decrease in ductility and increased processing difficulty, making the alloy prone to chipping and tool adhesion during conventional machining.

HEAs are widely regarded as typical difficult-to-machine materials due to their high melting point, high toughness, high strength, and high hardness. Research on their cutting performance and product realization remains in infancy, which limits the application of these materials in the engineering field [17,18]. To address the machining challenges of HEAs, some researchers have adopted non-traditional processing techniques such as wire electrical discharge machining (WEDM) to study their machinability. Mouralova et al. [13] first used WEDM to analyze the machinability of the newly developed HEAs (FeAsCrMnNNi and FeCoCrMnNiC_0.2_). Gunen et al. [19] machined MoNbTaTiZr refractory high-entropy alloys (RHEAs) using WEDM with brass wire electrodes and copper core electrodes. Zhang et al. [20] conducted WEDM experiments on four RHEAs with different constituent phases. The effects of constituent phases and processing parameters on the WEDM performance of RHEAs were investigated, and the relationship between the WEDM performance and the constituent phases of RHEAs was also established. Ceritbinmez et al. [21] machined HfNbTaTiZr RHEAs using WEDM and realized precision shaping of hard and brittle materials without subjecting them to high stresses. Chen et al. [22] investigated the WEDM performance of WNbMoTaZrx (x = 0.5, 1) RHEAs, finding that the cutting efficiency (CE) was significantly affected by the pulse-on time, pulse-off time, and peak current.

Electrical discharge machining (EDM) is a non-contact machining technology that operates on the same principle as WEDM, with both being based on the pulsed discharge erosion effect. During the discharge process, electrical energy is converted into thermal energy, which vaporizes or melts the workpiece surface material according to the shape of the forming electrode. Since the electrode and workpiece do not come into direct contact during processing, the cutting forces typically present in conventional machining are avoided. This enables high-precision machining and good surface quality, making EDM suitable for the fabrication of micro-holes with complex structures and high precision requirements. Giridharan et al. [23] investigated the influence of Inconel 718’s characteristics on the electric discharge drilling (EDD) process for micro-holes with a tubular brass electrode. The experimental results showed that the peak current plays a dominant role in the material removal rate (MRR), while pulse-on time dominates the hole taper. Nair et al. [24] investigated the surface characteristics of IN617 high-temperature alloy after EDM, focusing on the surface roughness, spattering area and white layer thickness. The experimental results showed that pulse-on time was the major factor affecting the spattering area and white layer thickness. To enhance the micro-hole precision in Super Ni 276, Saidulu et al. [25] focused on optimizing machining parameters, evaluating the influence of four key parameters: tool diameter, peak current, pulse-on time, and pulse-off time. The study compared the performance of brass and copper tools in EDD, focusing on MRR, electrode wear rate (EWR), recast layer thickness, heat-affected zone (HAZ), and micro-hardness distribution, emphasizing the importance of selecting appropriate tool materials to optimize EDD performance. To improve the EDM performance of micro-holes on the Ti-6Al-4V (TC4) titanium alloy, Zhang et al. [26] conducted a study on ultrasonic vibration-assisted EDM of micro-holes, investigating the mechanism of ultrasonic amplitude, and found that the optimal ultrasonic amplitude was 5.22 μm. Subsequently, Zhang et al. [27] carried out experimental investigations on machining performance under different machining parameters using the control variable method, revealing that ultrasonic vibration assistance can enhance the removal of eroded debris from the inter-electrode gap and improve discharge stability. Under the optimal process parameters, the MRR was increased by 4 times, and both the relative EWR and the micro-hole taper were reduced by approximately 50%. To solve the problem of strong parameter coupling in EDM of micro-holes on Hastelloy C-276 superalloy, Dutta et al. [28] achieved increased MRR, reduced tool wear and decreased diametral overcut by using response surface methodology and the multi-objective genetic algorithm. Taking SiCp/Al components with curved surfaces as the research object, Gong et al. [29] proposed a hybrid machining method combining micro-EDM and in situ mechanical reaming, realizing high-efficiency, low-wear, and recast-layer-free micro-hole machining. After process optimization, the MRR reached 0.069 mm^3^/min, the tool wear rate was 3.22%, and the surface roughness of the hole wall was reduced from Ra 1.452 μm to Ra 0.803 μm. For the difficult-to-machine material 17-4 PH stainless steel, Gerçekcioğlu et al. [30] carried out multi-response optimization for EDM by adopting Taguchi method-based gray relational analysis, achieving the collaborative optimization of multiple indicators, including surface roughness, MRR, and EWR. Recent studies have demonstrated that, besides electrical sensing detection, radio frequency (RF) monitoring in electrical discharge machining (EDM) is a low-cost, non-invasive detection method with high cost-effectiveness, which is convenient for practical engineering applications [31]. At present, Yao et al. have combined this monitoring technology with machine learning and interpretable artificial intelligence and realized the intelligent identification of discharge states in micro-electrical discharge machining (micro-EDM) [32]. This provides a promising and reliable process monitoring technology for micro-hole machining by EDM. In summary, EDM, as a non-contact precision machining technology, plays a crucial role in the micro-hole machining of difficult-to-machine materials such as high-temperature alloys, titanium alloys, and metal matrix composites [33], providing a valuable reference for exploring efficient and high-quality micro-hole machining of HEAs.

At present, research on HEAs’ machinability, especially special precision machining, is still relatively weak. The CoCrFeNiZr_0_._5_ EHEAs possess an FCC + Laves dual-phase structure, which leads to reduced plasticity and increased machining difficulty. Taking advantage of the non-contact and cutting-force-free characteristics of EDM, this paper first presents experimental research on the EDM of micro-holes in CoCrFeNiZr0.5 EHEAs using a tube electrode, realizing high-quality precision forming of micro-holes and filling the research gap in the field of EDM precision hole-making for such EHEAs. In EDM, previous studies have demonstrated that gap voltage, pulse-on time, pulse-off time, and pulse amplifier are the dominant influencing factors [27,30]. To comprehensively explore the influence mechanism of electrical parameters on micro-hole machining of HEAs, this paper investigates the effects of machining parameters, including gap voltage, pulse-on time, pulse-off time, and pulse amplifier, on machining speed, electrode wear, and machining quality. The intrinsic correlations among these factors are revealed, which offers a new method for the precision fabrication of micro-holes in EHEAs.

## 2. Experimental Setup and Materials

### 2.1. Experimental Materials

The CoCrFeNiZr_0.5_ EHEAs ingots were arc-melted by using high-purity raw metals (≥99.9 wt.%) under a Ti-guttered high-purity argon atmosphere without any heat treatment. To ensure composition uniformity, the mixed raw metals were flipped and re-melted six times in a water-cooled copper mold under electromagnetic stirring. Finally, the button ingots with a mass of 90 g and a thickness of approximately 5 mm were obtained. Then, the specimens were cut from the button ingots using WEDM (DK7735; Terui, Taizhou, China). The specimens were ground, polished, and etched for metallurgical observation.

**Figure 1:**
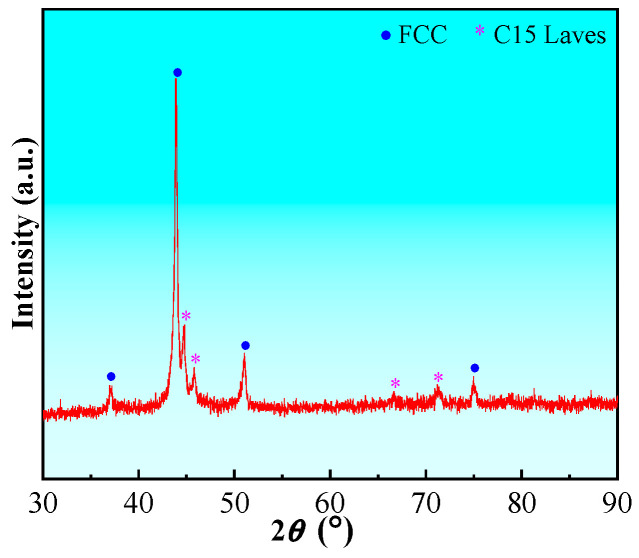
XRD pattern of the as-cast CoCrFeNiZr_0.5_ EHEAs.

The phase constitution of the CoCrFeNiZr_0.5_ EHEAs was identified by X-ray diffractometer (XRD; D8 Discover A25; Bruker, Karlsruhe, Germany) with a Cu-Kα target. A scanning step of 4°/min and a diffraction angle ranging from 30° to 90° were applied. The microhardness, HV, of the CoCrFeNiZr_0.5_ EHEAs was measured using a Vickers microhardness tester (401/402 MVD; Wilson Instruments, Norwood, MA, USA) with an applied load of 200 g and a dwell time of 15 s. At least 15 points at random places of the alloys were tested and the average value of Vickers microhardness was used. The microstructure and element distribution were carried out by scanning electron microscopy (SEM; CLARA; TESCAN, Brno, Czech Republic) equipped with an energy-dispersive spectrometer (EDS; Xplore 30; Oxford Instruments, Oxford, UK). Figure 1 displays the XRD pattern for as-cast CoCrFeNiZr_0.5_ EHEAs. The results clearly indicate that only two phases, i.e., FCC solid solution and the C15 Laves phase, are identified.

**Figure 2:**
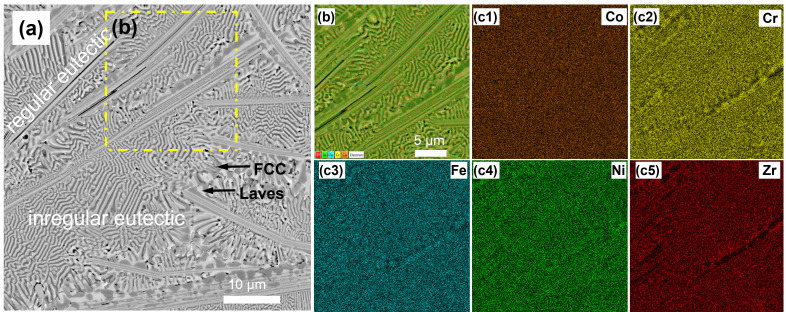
The BSE/SEM micrographs: (**a**) BSE micrograph; (**b**) enlarged image in dashed rectangular area; and (**c1**–**c5**) the corresponding EDS mapping showing the elemental distribution in the CoCrFeNiZr_0.5_ EHEAs.

The BSE/SEM micrographs showing the microstructure of as-cast CoCrFeNiZr_0.5_ EHEAs are shown in Figure 2a. A fully eutectic lamellar structure containing FCC phase and C15 Laves phase is clearly observed, which agrees well with the thermodynamic calculations [34]. It consists of alternatively irregular lamellar FCC and Laves phase. In addition, some regular FCC and Laves lamella are also found in the microstructure. During the solidification process, as the eutectic reaction occurs, the FCC phase and Laves phase grow in parallel with each other to form fine coupled lamella. The chemical compositions of the dark phase and bright phase were determined by EDS, as shown in Table 1. The dark phase contains 23.51 at.% Co, 25.5 at.% Cr, 25.31 at.% Fe, 19.67 at.% Ni, and 6.01 at.% Zr, which is identified as an FCC solid solution phase. The bright phase, corresponding to the Laves phase, comprises 23.37 at.% Co, 19.53 at.% Cr, 21.25 at.% Fe, 26.93 at.% Ni, and 8.93 at.% Zr. The enlarged micrograph in the dashed rectangular area and the corresponding EDS mapping of the CoCrFeNiZr_0.5_ EHEAs are shown in Figure 2b,c1–c5. In the CoCrFeNiZr_0.5_ EHEAs, the bright phases were enriched with Ni and Zr elements, indicating that the bright phases were a C15-type Laves phase, and the dark phases enriched with Fe and Cr were FCC solid solution phases. The microhardness of the present CoCrFeNiZr_0.5_ EHEAs is equal to 502 HV.

### 2.2. Micro-Hole EDM Machine

**Figure 3:**
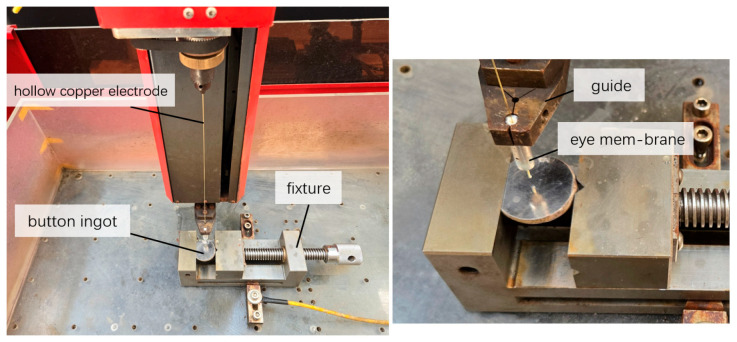
Photograph of the electric discharge drilling machine showing the button ingots, the hollow copper electrode, the guide, the eye membrane, and the fixture.

A CNC electric discharge drilling machine (EDDM) was used to process the prepared CoCrFeNiZr_0.5_ EHEAs button ingots. The experimental system consists of the machine tool, a working fluid circulation system, a power supply, and a motion control unit. The prepared CoCrFeNiZr_0.5_ EHEAs button ingot was installed in the fixture of the worktable, as shown in Figure 3, and its position was adjusted by the *x*-axis and *y*-axis of the machine tool. The tool electrode was a hollow brass wire with an outer diameter of 0.5 mm and an inner diameter of 0.2 mm. The hollow copper electrode was installed on the *Z*-axis and guided vertically downward through the eye membrane in the guide. During the processing, it rotated while feeding vertically downward. The positive pole and the negative pole of the pulse power supply were respectively connected to the EHEAs button ingot sample and the hollow copper electrode. The working fluid was pure water. The high-pressure pure water was delivered through the hollow copper electrode and directed to flow toward the EHEAs button ingot. When the distance between the hollow copper electrode and the button ingot sample reached the appropriate discharge gap, discharge machining began. The *Z*-axis continued to feed until the EHEA’s button ingot was penetrated. The high-pressure pure water flew through the hollow copper electrode and was sprayed into the discharge gap from the end of the copper electrode, adopting an internal flushing method to forcibly remove the discharge products.

According to the manual of the CNC EDDM, the recommended capacitor is 4 μF when the tool electrode diameter is 0.5 mm and 0.8 mm, and the other experimental parameters are shown in Table 2. The parameter values in Table 2 are the preliminary optimized range obtained from previous experiments.

**Figure 4:**
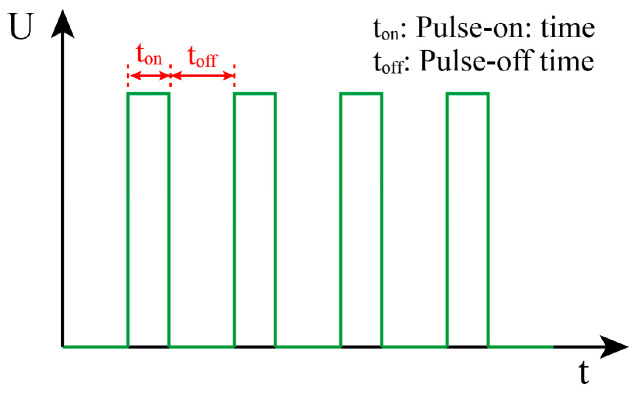
Schematic diagram of pulse parameters in EDM.

Based on this experimental scheme, multiple experiments were conducted. The effects of gap voltage, pulse-on time, pulse-off time, and pulse amplifier on the machining of micro-holes were analyzed. During processing, the time taken to machine the through-hole and the wear length of the hollow copper electrode were recorded. By comprehensively evaluating the machining time and the electrode wear length of the hollow copper electrode, gap voltage, pulse-on time, pulse-off time, and pulse amplifier were selected as the subsequent experimental parameters. It should be noted that the schematic diagram of the pulse parameter principle is shown in Figure 4, where pulse-on time refers to the duration of spark discharge between the tool electrode and the button ingot; pulse-off time refers to the interval between adjacent discharge pulses, which is used to restore the insulation of the discharge gap and remove the discharge debris.

The formulas adopted in this paper are specified as follows. The calculation formula for machining speed is given below:

$$v=\frac{H}{t}$$

In single-hole machining, *v* stands for machining speed with the unit of mm/min; *H* is the axial height of the cylindrical hole in mm; and *t* denotes the machining time in min.

The formula for calculating electrode wear is as follows:

$$l_{\theta }=L_{1}-L_{2}$$

In single-hole machining, $l_{\theta }$ is the electrode wear length (cm); $L_{1}$ is the electrode length before machining (cm); and $L_{2}$ is the electrode length after machining (cm).

The formula for calculating taper is as follows:

$$K=\frac{D_{b}-D_{s}}{H}$$

where *K* refers to taper, a dimensionless parameter that reflects the variation rate of diameter along the axial direction; $D_{b}$ is the major diameter of the cylindrical hole (μm); $D_{s}$ is the minor diameter of the cylindrical hole (μm); *H* is the axial height of the cylindrical hole (μm).

## 3. Results and Discussion

### 3.1. Machining Efficiency and Electrode Wear

#### 3.1.1. The Influence of Gap Voltage

**Figure 5:**
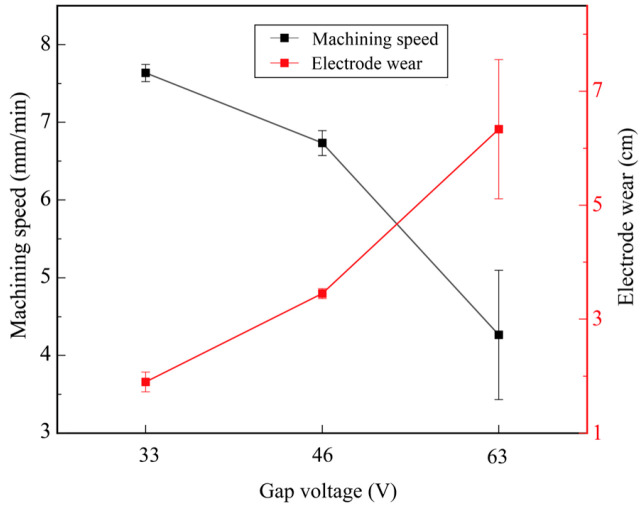
Effect of gap voltage on machining efficiency and electrode wear.

Figure 5 shows the effect of the gap voltage on machining efficiency and electrode wear. The other parameters were set as follows: pulse-on time of 5 μs, pulse-off time of 3 μs, and pulse amplifier of 5 A. When the gap voltage increased from 33 V to 63 V, the machining speed decreased from 7.63 mm/min to 4.26 mm/min. At a gap voltage of 63 V, the machining speed exhibited a sharp change, with a standard deviation of 0.83. This phenomenon occurs because the increase in gap voltage enhances the single-pulse discharge energy [27]. The larger the single-pulse discharge energy is, the larger the particle size of discharge debris will be. Given the narrow discharge gap in micro-hole machining, it is difficult to remove such large debris from the discharge gap in a timely manner, and the accumulated debris significantly increases the risk of abnormal discharge and short circuits [26]. Consequently, the electrode oscillates repeatedly, which reduces the machining stability and further results in a decrease in machining efficiency.

With the increase in gap voltage, electrode wear also showed an upward trend, rising rapidly from 1.90 cm to 6.33 cm. At 63 V, a large standard deviation of electrode wear was also observed. Similarly to the mechanism that affects machining efficiency, large discharge debris in the narrow discharge gap cannot be discharged in a timely manner, resulting in debris accumulation inside the discharge gap. Excessively accumulated debris will induce abnormal discharge and further aggravate electrode wear [26].

#### 3.1.2. The Influence of Pulse-On Time

**Figure 6:**
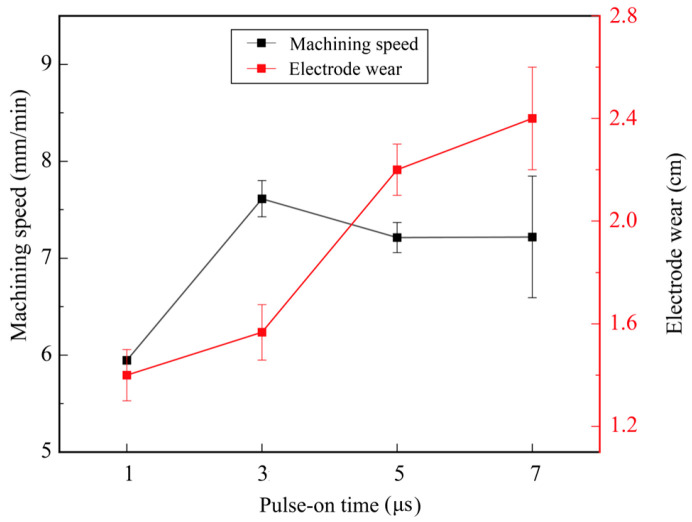
Effect of pulse-on time on machining efficiency and electrode wear.

Figure 6 shows the effect of pulse-on time on machining efficiency and electrode wear. The other parameters were set as follows: gap voltage of 33 V, pulse-off time of 3 μs, and pulse amplifier of 5 A. When the pulse-on time increased from 1 μs to 3 μs, the machining efficiency increased from 5.95 mm/min to 7.61 mm/min. At 7 μs, the machining efficiency decreased to 7.22 mm/min. At low pulse-on time, an increase in pulse-on time raises the discharge energy, thereby improving machining efficiency. However, when the pulse-on time exceeds 3 μs, excessive discharge energy will generate large debris particles. The debris that cannot be discharged in a timely manner leads to process instability and reduces machining efficiency [27]. The longer the pulse-on time, the more pronounced the machining instability becomes, which is consistent with the large standard deviation in machining efficiency observed at 7 μs.

As the pulse-on time increases from 1 μs to 7 μs, the electrode wear shows an upward trend, increasing from 1.40 cm to 2.4 cm. According to the research by Gerçekcioğlu et al. [30], an increase in pulse-on time gives rise to larger discharge debris. Large discharge debris increases the probability of secondary discharge occurring on the electrode, thus leading to more severe electrode wear. Meanwhile, this process is accompanied by repeated oscillatory feeding of the electrode, which further exacerbates the electrode wear.

#### 3.1.3. The Influence of Pulse-Off Time

**Figure 7:**
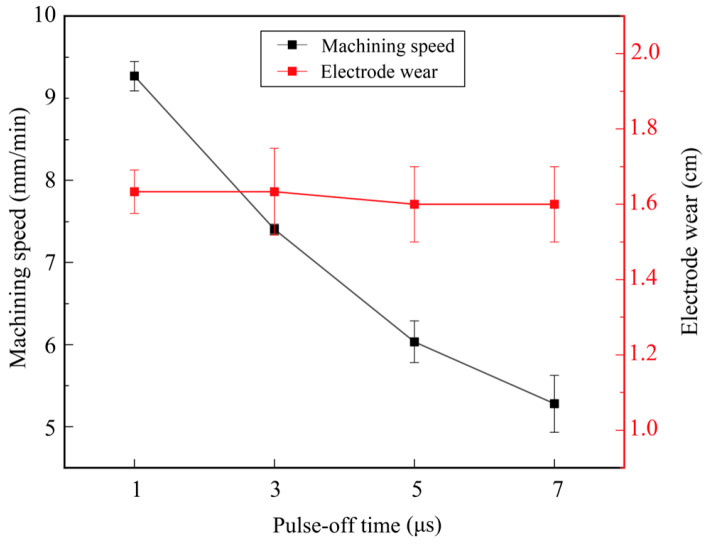
Effect of pulse-off time on machining efficiency and electrode wear.

Figure 7 shows the effect of pulse-off time on machining efficiency and electrode wear. The other parameters were set as follows: gap voltage of 33 V, pulse-on time of 3 μs, and pulse amplifier of 5 A. As the pulse-off time increased from 1 μs to 7 μs, the machining speed decreased from 9.27 mm/min to 5.38 mm/min. With the increase in pulse-off time, the discharge energy density per unit time decreases, which extends the overall machining duration and further reduces the machining speed. With the variation in pulse-off time, the electrode wear varies slightly and remains between 1.60 cm and 1.63 cm. This indicates that under this set of parameters, the pulse-off time is adequate to restore the insulation performance of the discharge gap and evacuate the eroded products, thereby ensuring machining stability. As a result, the electrode wear remains relatively stable.

#### 3.1.4. The Influence of Pulse Amplifier

**Figure 8:**
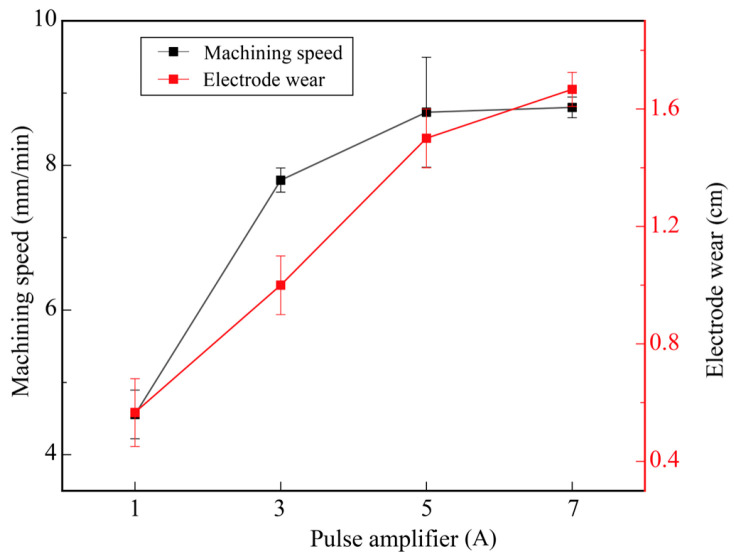
Effect of pulse amplifier on machining efficiency and electrode wear.

Figure 8 shows the effect of pulse amplifier on machining efficiency and electrode wear. The other parameters were set as follows: gap voltage of 33 V, pulse-on time of 3 μs, and pulse-off time of 1 μs. When the pulse amplifier increased from 1 A to 7 A, the machining speed increased from 4.56 mm/min to 8.80 mm/min. However, the increasing trend gradually slowed down, with the increase in amplitude dropping from 3.24 mm/min (from 1 A to 3 A) to 0.07 mm/min (from 5 A to 7 A). It can be observed that as the pulse amplifier increases, the discharge energy gradually increases, leading to an improvement in the machining speed. When the pulse current rises to a certain value, the machining efficiency and debris removal efficiency gradually reach an equilibrium state. Limited by the insufficient debris removal capacity during micro-hole machining, the machining efficiency no longer increases significantly and remains stable at approximately 8.8 mm/min. When the pulse amplifier increased from 1 A to 7 A, the electrode wear rose from 0.57 cm to 1.67 cm, indicating that as the pulse amplifier increases, the discharge becomes more intense, the probability of abnormal discharge increases, and the electrode wear rises [26]. Based on the above single-factor experimental results, the optimal micro-hole machining parameters are determined by comprehensively considering machining efficiency and electrode wear: gap voltage of 33 V, pulse-on time of 3 μs, pulse-off time of 1 μs, and pulse amplifier of 3 A. Adopting the parameters to process a button ingot sample with a depth of 5 mm, the machining speed is 7.79 mm/min, and the electrode wear is 1 cm.

### 3.2. Metallographic Observation of the Machined Micro-Holes

According to Figure 5, Figure 6, Figure 7 and Figure 8, the machining speed ranged from 4 to 9 mm/min, and the gap voltage had the greatest influence on electrode wear. To further observe the morphology of the machined micro-holes, micro-holes were machined under a pulse-on time of 5 μs, a pulse-off time of 3 μs, a pulse amplifier of 5 A, and a gap voltage ranging from 33 V to 63 V. The specimens were ground using sandpapers from 180# to 2000# and then polished using a polishing machine. After being etched in a mixed solution of HCl:HNO_3_:H_2_O = 3:1:2 for 15 s, the specimens were used for microstructural observation. The microstructure and element distribution were characterized by using Hexagon’s laser confocal microscope and scanning electron microscopy (SEM; CLARA; TESCAN, Brno, Czech Republic) equipped with an energy-dispersive spectrometer (EDS; Xplore 30).

**Figure 9:**
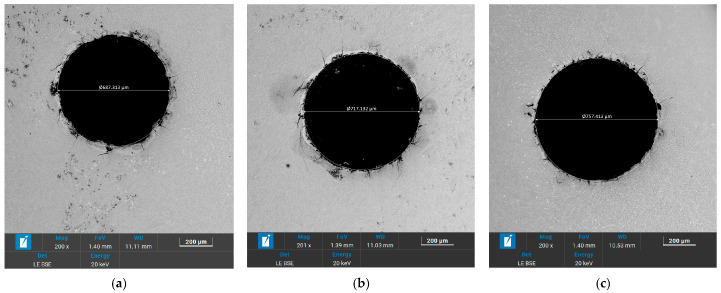
Micro-holes machined under different gap voltages: (**a**) 33 V; (**b**) 46 V; (**c**) 63 V.

Figure 9 shows the micro-holes morphology machined under different gap voltages. For all voltages, a recast layer and micro-cracks were present at the edges of the hole, which are common characteristics of EDM-machined surfaces. At the gap voltages of 33 V, 46 V and 63 V, the corresponding diameters of holes were 687.313 μm, 717.132 μm, and 757.413 μm. It can be observed that the diameters of holes increase with increasing gap voltage. This is consistent with the findings discussed in Section 3.1.1: as the gap voltage increases, the discharge energy increases, leading to a larger discharge gap and consequently a larger hole diameter. Therefore, using a lower gap voltage is advantageous for machining smaller-diameter holes.

**Figure 10:**
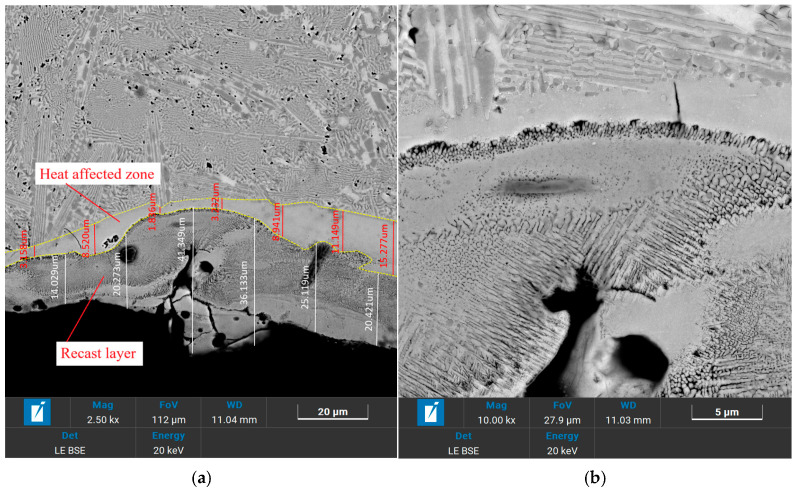
Magnified micrograph of the micro-hole machined at a gap voltage of 33 V; (**a**) thickness of the recast layer; (**b**) thickness of the HAZ.

The micro-hole machined at a gap voltage of 33 V was magnified, as shown in Figure 10. The uppermost layer of the machined surface is the recast layer, the middle layer is the HAZ, and the inner layer is the base material. The boundaries between the three layers are distinct. High-magnification observation reveals that the thickness of the recast layer ranges from 14.029 μm to 41.349 μm, with an average value of 26.220 μm, and it is mainly composed of columnar grains and equiaxed grains. The thickness of HAZ is between 1.836 μm and 15.277 μm, with an average of 7.445 μm, which primarily consists of fine equiaxed grains. Furthermore, defects such as micro-cracks and voids are primarily present in the recast layer and the HAZ, indicating that the EDM of micro-holes in HEAs can control defect propagation and maintain the good performance of the base material.

**Figure 11:**
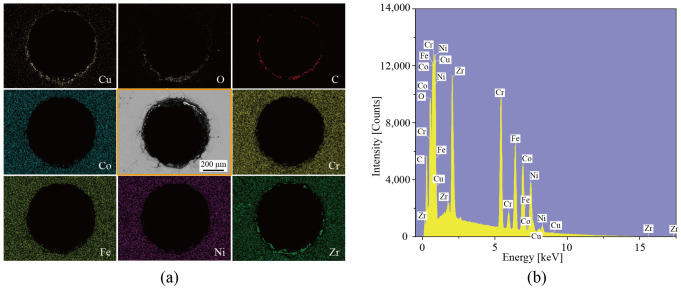
EDS elemental map of the micro-hole machined at a gap voltage 33 V; (**a**) EDS; (**b**) elemental analysis.

EDS was adopted to detect the elemental composition on the machined cross-section. The micro-hole machined at a gap voltage of 33 V was selected for area-scan analysis, and the results are shown in Figure 11a, with the corresponding elemental analysis presented in Figure 11b. The specimen as a whole remains rich in Co, Cr, Fe, Ni, and Zr, which are the constituent elements of the CoCrFeNiZr_0.5_ EHEAs. However, Cu, O, and C are mainly present in the recast layer and the HAZ, indicating that high-temperature melting occurred on the material surface during machining and that Cu from the electrode and C from the dielectric medium intermixed with the HEAs to form the recast layer. In addition, oxidation reactions occurred on the workpiece surface at high temperatures, resulting in an enrichment of O in the recast layer and the HAZ.

**Figure 12:**
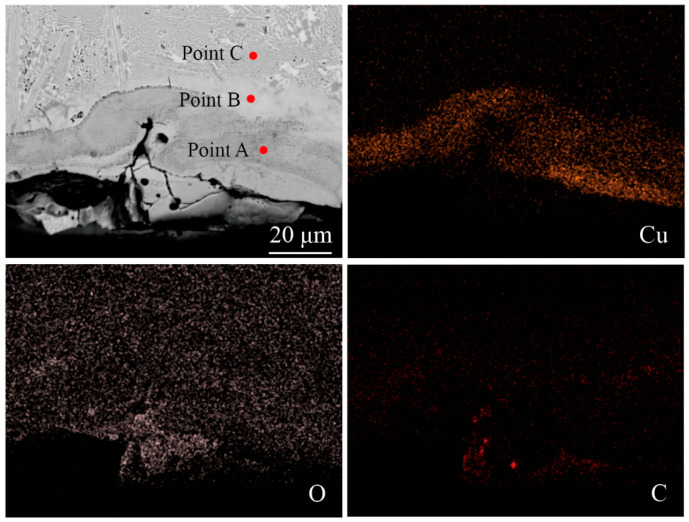
EDS elemental distribution of the local region.

To precisely quantify the elemental differences between the recast layer and the base material, a local region in Figure 10a was selected for further elemental analysis. The area-scan results are shown in Figure 12, where it can be more clearly observed that elements such as Cu and O are mainly concentrated in the recast layer.

Three typical points, Point A, Point B, and Point C in Figure 12, were selected for point EDS analysis, and the results are presented in Table 3. In the recast layer, the content of Cu is 10.88%, the content of O is 4.02%, and the content of C is 12.06%, all of which are the highest among the three points. The contents of these three elements in the HAZ are lower than those in the recast layer. In the base material, no Cu is detected, and the trace amounts of O and C may originate from the air and the polishing agent. The elemental analysis of the local features further indicates that the high-temperature erosion occurs only on the surface region of the workpiece, while the base material retains its original metallic properties.

**Figure 13:**
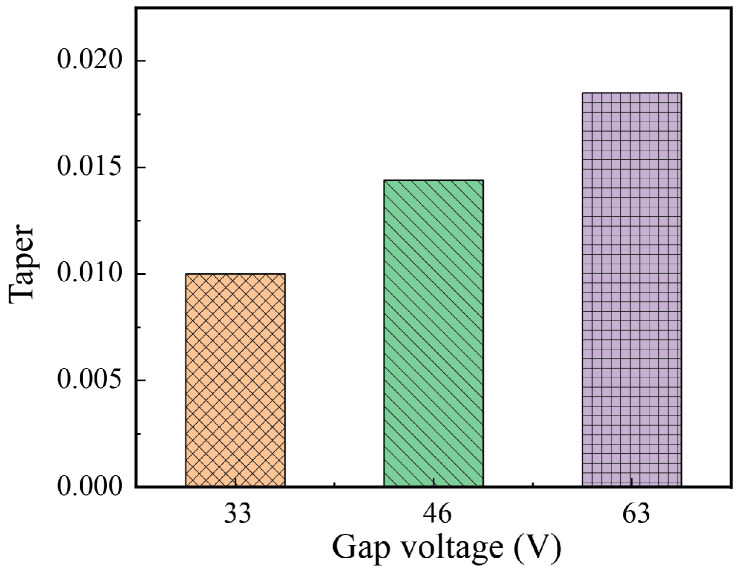
Hole taper under different voltages.

The taper of micro-holes machined under different voltages is shown in Figure 13. With the increase in gap voltage, the micro-hole taper increases accordingly, rising from 0.0100 at 33 V to 0.0185 at 63 V. As analyzed in Section 3.1.1, the elevation of gap voltage will increase the size of discharge debris. The accumulation of large-sized discharge debris in the machining gap is prone to inducing abnormal discharge, which repeatedly performs electric discharge machining on the hole wall. This leads to a continuous expansion of the inlet diameter during the machining process and ultimately increases the micro-hole taper.

## 4. Conclusions

This paper investigates the EDM micro-hole machining performance of CoCrFeNiZr_0.5_ EHEAs and explores its application potential in high-value component manufacturing. By analyzing the material’s inherent characteristics and optimizing EDM parameters, high-quality micro-holes were successfully machined. The microstructure and elemental distribution of the machined holes were systematically analyzed. The main conclusions are summarized as follows:

(1) The EDM micro-hole drilling in CoCrFeNiZr_0.5_ EHEAs was investigated, which renders an efficient and precision machining capability for enhanced engineering design and manufacture of HEAs and their high-value engineering applications.
(2) The effects of the EDM parameters were investigated, including the gap voltage, pulse-on time, pulse-off time, and pulse amplifier, on machining speed and electrode wear, and process optimization was achieved. The optimal micro-hole machining parameters were determined by comprehensively considering machining efficiency and electrode wear: gap voltage of 33 V, pulse-on time of 3 μs, pulse-off time of 1 μs, and pulse amplifier output of 3 A. Adopting the parameters to process a button ingot sample with depth of 5 mm, the machining speed is 7.79 mm/min and electrode wear is 1 cm.
(3) Elemental analysis of the micro-hole’s microstructure revealed that surface material underwent melting, and elements such as Cu, O, and C were enriched in the recast layer but not significantly present in the base material. This demonstrates that EDM micromachining of HEAs can preserve the original metallic properties of the base material and make it a stable and reliable machining method.
(4) Work Prospect: As a non-intrusive and cost-effective discharge state recognition method, RF signal monitoring will be integrated into a self-developed EDM micro-hole machining platform in follow-up research. Accurate recognition of various discharge states will be realized through the characteristics of voltage and current signals, providing technical support for the efficient and stable machining of EHEAs micro-holes.

## Figures and Tables

**Table 1:** EDS results in different phases of the CoCrFeNiZr_0.5_ EHEAs (at.%).

Region	Co	Cr	Fe	Ni	Zr
FCC phase	24.51	26.5	25.31	19.67	4.01
Laves phase	23.37	19.53	21.25	26.93	8.93

**Table 2:** Experimental parameters.

Parameters	Value
Gap Voltage (V)	33, 46, 63
Pulse-on time (μs)	1, 3, 5, 7
Pulse-off time (μs)	1, 3, 5, 7
Pulse Amplifier (A)	1, 3, 5, 7
Pressure of working fluid (MPa)	10
Wire rotating speed (r/min)	100

**Table 3:** Element distribution of Point A, Point B and Point C.

Element	Mass% of Point A	Mass% of Point B	Mass% of Point C
Cu	10.88 ± 0.09	0.21 ± 0.03	nd
O	4.02 ± 0.04	1.62 ± 0.03	0.68 ± 0.02
C	12.06 ± 0.05	9.68 ± 0.05	3.56 ± 0.03
Cr	14.22 ± 0.07	17.84 ± 0.08	28.69 ± 0.10
Fe	18.09 ± 0.08	18.23 ± 0.08	26.67 ± 0.11
Co	19.43 ± 0.10	19.80 ± 0.10	22.39 ± 0.11
Ni	17.92 ± 0.10	19.57 ± 0.11	15.62 ± 0.10
Zr	3.38 ± 0.05	13.06 ± 0.08	2.39 ± 0.05
Otal	100.00	100.00	100.00

## Data Availability

The original contributions presented in this study are included in the article. Further inquiries can be directed to the corresponding author.
